# Quantifying the individual impact of artificial barriers in freshwaters: A standardized and absolute genetic index of fragmentation

**DOI:** 10.1111/eva.13044

**Published:** 2020-07-28

**Authors:** Jérôme G. Prunier, Camille Poesy, Vincent Dubut, Charlotte Veyssière, Géraldine Loot, Nicolas Poulet, Simon Blanchet

**Affiliations:** ^1^ Centre National de la Recherche Scientifique (CNRS) Université Paul Sabatier (UPS) UMR 5321 Station d’Ecologie Théorique et Expérimentale Moulis France; ^2^ CNRS IRD Avignon Université IMBE Aix Marseille Univ Marseille Université France; ^3^ CNRS UPS UMR 5174 EDB (Laboratoire Évolution & Diversité Biologique) École Nationale de Formation Agronomique (ENFA) Toulouse Cedex 4 France; ^4^ DRAS, Pôle R&D écohydraulique OFBIMFT‐PPRIME Office Français de la Biodiversité Toulouse France

**Keywords:** bio‐indicator, dams, fragmentation, freshwater fish, genetic differentiation, riverscape connectivity, simulations, weirs

## Abstract

Fragmentation by artificial barriers is an important threat to freshwater biodiversity. Mitigating the negative aftermaths of fragmentation is of crucial importance, and it is now essential for environmental managers to benefit from a precise estimate of the individual impact of weirs and dams on river connectivity. Although the indirect monitoring of fragmentation using molecular data constitutes a promising approach, it is plagued with several constraints preventing a standardized quantification of barrier effects. Indeed, observed levels of genetic differentiation *GD* depend on both the age of the obstacle and the effective size of the populations it separates, making comparisons of the actual barrier effect of different obstacles difficult. Here, we developed a standardized genetic index of fragmentation (*F*_INDEX_), allowing an absolute and independent assessment of the individual effects of obstacles on connectivity. The *F*_INDEX_ is the standardized ratio between the observed *GD* between pairs of populations located on either side of an obstacle and the *GD* expected if this obstacle completely prevented gene flow. The expected *GD* is calculated from simulations taking into account two parameters: the number of generations since barrier creation and the expected heterozygosity of the populations, a proxy for effective population size. Using both simulated and empirical datasets, we explored the validity and the limits of the *F*_INDEX_. We demonstrated that it allows quantifying effects of fragmentation only from a few generations after barrier creation and provides valid comparisons among obstacles of different ages and populations (or species) of different effective sizes. The *F*_INDEX_ requires a minimum amount of fieldwork and genotypic data and solves some of the difficulties inherent to the study of artificial fragmentation in rivers and potentially in other ecosystems. This makes the *F*_INDEX_ promising to support the management of freshwater species affected by barriers, notably for planning and evaluating restoration programs.

## 1 INTRODUCTION

Heavily impacted by human activities, rivers are at the heart of biodiversity conservation issues (Dudgeon et al., 2006; Reid et al., 2018). Among the various threats to these ecosystems, river fragmentation by artificial barriers is considered as the most widespread and worrying (Couto & Olden, 2018; Nilsson, 2005; Turgeon, Turpin, & Gregory‐Eaves, 2019). Weirs and dams, but also pipes and culverts, have long been, and are still, constructed for flow regulation and/or hydropower supply but they often imply a loss of habitat and a reduction in riverscape functional connectivity (that is, species‐specific) in freshwater organisms (Birnie‐Gauvin, Aarestrup, Riis, Jepsen, & Koed, 2017; Jansson, Nilsson, & Malmqvist, 2007). For fish, artificial fragmentation is known to impact key biological processes such as migration, dispersal, and recruitment, and thus viability and productivity of populations and communities (Blanchet, Rey, Etienne, Lek, & Loot, 2010; Poulet, 2007; Turgeon et al., 2019). Given the central role of hydropower as a source of energy, mitigating these negative aftermaths is now of high importance (Couto & Olden, 2018; Gibson, Wilman, & Laurance, 2017).

Different restoration and mitigation measures may be considered to enhance longitudinal river connectivity, including the removal of obstacles, periodic turbine shutdowns, and fishpasses setting (Bednarek, 2001; Poff & Schmidt, 2016; Silva et al., 2018). However, these measures may all result in unintended outcomes (McLaughlin et al., 2013), or unsatisfactory trade‐offs between conservation of biodiversity, preservation of historical and cultural legacy and the maintenance of services provided by obstacles (Gibson et al., 2017; Hand et al., 2018; Roy et al., 2018; Song et al., 2019). In terms of conservation planning, it is therefore essential that environmental managers benefit from precise estimates of the actual impacts of different obstacles on river connectivity, or from precise estimates of the gain in connectivity resulting from restoration actions, in order to guide the prioritization of conservation efforts and to evaluate their efficiency (Cooke & Hinch, 2013; Januchowski‐Hartley, Diebel, Doran, & McIntyre, 2014; Raeymaekers, Raeymaekers, Koizumi, Geldof, & Volckaert, 2009).

The direct monitoring methods conventionally used in rivers to quantify the functional permeability of an obstacle or the efficiency of a restoration action are video counting, telemetry, and capture–recapture protocols. Although efficient (Cooke & Hinch, 2013; Hawkins, Hortle, Phommanivong, & Singsua, 2018; Junge, Museth, Hindar, Kraabøl, & Vøllestad, 2014; Pracheil, Mestl, & Pegg, 2015), these methods are associated with technical constraints. In particular, ecological studies based on video counting or telemetry are often conducted on a limited number of obstacles, whereas robust capture–recapture protocols imply repeated and exhaustive capture sessions, ideally over several years, which involves the mobilization of substantial human and financial resources (Cayuela et al., 2018).

Indirect monitoring based on molecular data constitutes a promising alternative approach, allowing multi‐specific studies of dam‐induced fragmentation (Selkoe, Scribner, & Galindo, 2015). Among the many analytical procedures developed in recent years to quantify the mobility of organisms on the basis of genetic or genomic data, assignment methods, and parentage analyses (Jombart, Devillard, & Balloux, 2010; Pritchard, Stephens, & Donnelly, 2000; Städele & Vigilant, 2016; Wilson & Rannala, 2003) allow the detection of “real‐time” noneffective movements (i.e., not necessarily followed by a reproduction event; e.g., Junge et al., 2014; Raeymaekers et al., 2009; Saint‐Pé et al., 2018) but they usually require an extensive sampling of individuals and moderate to high genetic differentiation between populations (Broquet & Petit, 2009; Cayuela et al., 2018).

An alternative method to quantify the permeability of an obstacle from molecular data is simply to measure the level of neutral genetic differentiation between populations located in the immediate upstream and downstream vicinity of an obstacle (i.e., located a few hundreds of meters to one kilometer apart, an *adjacent sampling strategy*), an approach that does not necessarily require large sample sizes (i.e., *n* ~ 20–30 per population) or heavy computation: Any drop in local functional connectivity due to the creation of a barrier to gene flow is expected to translate into an increase in neutral genetic differentiation (Raeymaekers et al., 2009). However, measures of neutral genetic differentiation may only be considered as correct estimates of actual barrier effects when comparing obstacles of the *same* age (in terms of number of generations since barrier creation) and/or separating populations of *similar* effective size. This is because genetic differentiation primarily stems from genetic drift, that is, from the random fluctuation of allelic frequencies naturally occurring in all populations (Allendorf, 1986). When populations are separated by an obstacle to gene flow, these fluctuations tend to occur independently in each population, leading to a differential distribution of allelic frequencies on either side of the barrier. However, this process is progressive, taking place over several generations (Landguth et al., 2010), and is all the more slow as effective population sizes *N_e_* are large (Kimura, 1983). As a consequence, it is impossible to attribute the differences in levels of genetic differentiation observed between obstacles varying in age and/or in the effective size of populations they separate to differences in their actual barrier effects; older obstacles or obstacles separating smaller populations should show higher genetic differentiation than more recent obstacles or obstacles separating larger populations, despite similar actual barrier effects. Given this drawback, there is an urgent need for the development of a *standardized* and *absolute* genetic index of fragmentation that takes into account the contribution of both the age of the obstacle (expressed in the number of generations since barrier creation) and the effective size *N_e_* of populations (or a proxy of it since this parameter is notoriously difficult to quantify; Wang, 2005) to observed measures of genetic differentiation. Such an index might allow a quick and robust quantification of individual and actual barrier effects whatever their characteristics, paving the way for informed management prioritization and proper evaluation of restoration measures, along with inter‐basins and interspecific comparative studies.

Here, we bridge that gap by developing a user‐friendly and standardized genetic index of fragmentation (see Appendix S1 for a walkthrough), allowing an absolute and independent assessment of the individual effects of obstacles on gene flow. The proposed index (*F*_INDEX_) is expressed as a percentage and directly quantifies the relative loss of gene flow resulting from the presence of an obstacle. It is based on the comparison of measures of genetic differentiation observed between populations located in the immediate upstream and downstream vicinity of a putative obstacle with the theoretical measures of genetic differentiation that would be expected if the obstacle was a total barrier to gene flow. These theoretical measures of genetic differentiation are inferred from numerous genetic simulations, here used to reflect the expected changes in allelic frequencies resulting from the interplay between the age of the obstacle and the expected heterozygosity of populations, a proxy for *N_e_*: the closer the observed measure of genetic differentiation from the one that would be expected in the worst‐case scenario (total barrier to gene flow), the higher the index of fragmentation. We first present the logic and principles underlying our index. We then use both simulated and published empirical genetic datasets to explore and discuss the validity and the limits of the proposed index. We finally propose several perspectives to use the index and, because setting bio‐indicators takes time, we present potential improvements that should be considered to make this index even more useful to managers.

## 2 MATERIALS AND METHODS

### 2.1 Principle of the proposed genetic index of fragmentation *F*_INDEX_

The proposed genetic index of fragmentation *F*_INDEX_ is designed as a standardized estimate of the reduction in gene flow between two adjacent populations separated by an obstacle. It simply consists in rescaling the observed measure of genetic differentiation GD_obs_ within its theoretical range of variation, taking into account the expected temporal evolution of allelic frequencies resulting from the interplay between the age of the obstacle and the averaged expected heterozygosity of populations, a proxy for their effective population size *N_e_*. This theoretical range of variation spans from GD_min_ to GD_max_. GD_min_ stands for the theoretical measure of genetic differentiation that would be expected if the obstacle was totally permeable to gene flow (crossing rate *m* ≈ 0.5). GD_min_ should theoretically equal 0 but the background noise resulting from the concomitant influences of genetic drift, mutations, and incomplete genetic sampling may actually lead to non‐null—though very low—measures of genetic differentiation. On the other hand, GD_max_ stands for the theoretical measure of genetic differentiation that would be expected under the worst‐case scenario, that is, under the hypothesis that the considered obstacle is a total barrier to gene flow (*m* = 0). GD_max_ is expected to increase with time since barrier creation and to decrease with the increase in effective population sizes (Gauffre, Estoup, Bretagnolle, & Cosson, 2008; Landguth et al., 2010) and thus with *H_e_*. For any measure *k* of genetic differentiation GD*^k^*, the genetic index of fragmentation *F*_INDEX_ is then computed as follows (see Appendix S2 for details):

(1) $$F_{\text{INDEX}}=\left(\frac{\ln\left({\text{GD}}_{\min}^{k}\right)‐\ln\left({\text{GD}}_{\text{obs}}^{k}\right)}{\ln\left({\text{GD}}_{\min}^{k}\right)‐\ln\left({\text{GD}}_{\max}^{k}\right)}\right)\times 100$$

The *F*_INDEX_ ranges from 0% (the observed measure of genetic differentiation is minimum—but not null—and equals the expected value GD_min_ under the assumption that the considered obstacle has no impact on gene flow) to 100% (the observed measure of genetic differentiation is maximum and equals the expected value GD_max_ under the assumption that the considered obstacle acts as a total barrier to gene flow). The *F*_INDEX_ thus directly quantifies the loss of gene flow resulting from the presence of an obstacle.

GD_obs_ is directly calculated from observed genotypic data collected in populations located at the immediate upstream and downstream vicinity of the obstacle (a few hundred of meters to one kilometer apart depending on the target species; see below), whereas GD_min_ and GD_max_ are predicted from theoretical datasets simulated according to three main parameters (see the next section for details): the mutation rate *µ* of considered genetic markers, and, for GD_max_ only, the age T of the total barrier to gene flow (expressed in number of generations since barrier creation; Landguth et al., 2010; Lowe & Allendorf, 2010) and the averaged expected heterozygosity *H_e_* of the two considered populations. *H_e_* is here considered as a proxy for effective population sizes *N_e_*, since both theoretical and empirical works indicate that genetic diversity should increase with the increase in *N_e_* (Hague & Routman, 2016; Kimura, 1983; see Appendix S5). We used the average of expected levels of heterozygosity since most pairwise metrics of genetic differentiation assume similar effective population sizes between populations (Prunier, Dubut, Chikhi, & Blanchet, 2017).

### 2.2 Expected measures of genetic differentiation

We used QuantiNemo2 (Neuenschwander, Michaud, & Goudet, 2019), an individual‐based simulator for population genetics, to simulate theoretical datasets that will in turn be used to predict GD_min_ and GD_max_ values. We designed a very simple meta‐population model composed of two adjacent demes. Both demes had the same carrying capacity *K*, with *K* ranging from 30 to 2,000 individuals (93 levels; see Figure S5a for visualization) and kept constant over time. We used forward simulations of gene flow between these two demes over 1,000 nonoverlapping generations. Genetic polymorphism was based on 15 microsatellite loci and 20 alleles per locus, which corresponds to the number of markers typically used in empirical studies focusing on functional connectivity (Blanchet et al., 2010; Coleman et al., 2018; Storfer, Murphy, Spear, Holderegger, & Waits, 2010). The mutation rate *µ*, following a stepwise mutation model, was set to 5 × 10^–5^ or 5 × 10^–4^, so as to explore the natural variability observed in microsatellite markers (mutation rate ranging from 10^–6^ to 10^–2^; Li, Korol, Fahima, Beiles, & Nevo, 2002; Schlötterer, 2000; Yue, David, & Orban, 2007). Genotypes were randomly assigned to individuals at the beginning of simulations. The inter‐deme migration rate was set to 0.5 for the first 400 generations, these parameters providing an optimal mixing of populations and mimicking a natural situation without barrier. The inter‐deme migration rate was then dropped to zero for the last 600 generations, mimicking the creation of a total barrier to gene flow, splitting a “single” population into two adjacent subpopulations. With populations being isolated for 600 generations, we made sure our simulations covered a time frame long enough to account for the effect of the oldest artificial barriers: Although most obstacles in freshwater ecosystems around the world are recent (constructed over the last 100 years), many others, especially in Europe, date from the 12th–15th centuries, which corresponds to ~250–400 generations in most aquatic organisms such as fish species (assuming a generation time of 2 years). For each carrying capacity *K* (93 levels) and each mutation rate *µ* (2 levels), we ran ten simulation replicates, and 30 genotypes were sampled every ten generations from generation 300 to generation 1,000 (71 levels) to monitor the setting up of genetic differentiation over time. This procedure resulted in a total of 93 × 2×71 × 10=132,060 simulated genetic datasets in the *Fstat* format (Goudet, 1995) and further converted into the *genepop* format (Rousset, 2008) using R (R Development Core Team, 2014).

For each simulation, we computed the two following pairwise metrics of genetic differentiation: the Hedrick's G″st (Hedrick, 2005; Meirmans & Hedrick, 2011) and the Meirmans’ φ′st (Meirmans, 2006), both computed using the R‐package *mmod* (Winter, 2012). Nine other metrics were initially considered, but preliminary analyses revealed that some were dependent on sample size (e.g., the proportion of shared alleles or the Cavalli‐Sforza and Edwards’ Chord distance; Bowcock et al., 1994; Cavalli‐Sforza & Edwards, 1967; see Appendix S3 for details), while others were sensitive to mutation rate and/or did not show enough variability (e.g., the Weir and Cockerham's θst or the Jost's D; Jost, 2008; Weir & Cockerham, 1984; see Appendix S4 for details): They were thus discarded to avoid jeopardizing the validity of the proposed index. We found that the two retained metrics G″st and φ′st were robust to variations in mutation rate and increased quickly after barrier creation, especially in the case of small effective population sizes (Appendix S4), in accordance with theoretical expectations (Lowe & Allendorf, 2010; Meirmans & Hedrick, 2011). All negative G″st and φ′st values were set to 0. For each simulated dataset, we also computed the averaged expected heterozygosity *H_e_* over the 15 loci in each population. *H_e_* was then averaged over the two populations and further considered as a proxy for effective population sizes (see Appendix S5a). *H_e_* increased monotonically with carrying capacity in our simulations, in accordance with both theoretical and empirical works (Hague & Routman, 2016; Kimura, 1983). We here focused on mean heterozygosity because, unlike metrics such as allelic richness, heterozygosity values are bound between 0 and 1, which facilitates comparison between case studies. Moreover, this metric is much more straightforward to calculate for managers than the actual effective population size, since the latter is notoriously difficult to estimate in complex landscapes (Paz‐Vinas et al., 2013; Wang, 2005). Note also that the use of two different realistic mutation rates yielded two levels of *H_e_* across simulations (a low level at the low mutation rate and a high level at the high mutation rate; Appendix S5b), thus mimicking uncertainty in our proxy for effective population sizes. In addition to the two metrics of genetic differentiation G″st and φ′st and to the expected heterozygosity *H_e_*, we also kept record of the simulation replicate number, the mutation rate *µ*, the generation *t* at which genotypes were collected, the age *T* of the barrier (computed as *T* = *t*−400 and expressed in number of generations since barrier creation), and the carrying capacity *K* of simulated populations.

The 111,600 simulations associated with *T* > 0 (i.e., after the creation of the barrier) were used as a training set in the regression implementation of a random forest machine‐learning algorithm (Breiman, 2001). This approach was chosen as it is currently one of the most efficient statistical techniques for making predictions from nonlinear data, with only a few parameters to tune (Genuer, Poggi, Tuleau‐Malot, & Villa‐Vialaneix, 2017). The objective was to establish theoretical distributions of G″st and φ′st allowing future predictions of GD_max_ values according to both *T* and *H_e_*. For each mutation rate *µ* and each metric of genetic differentiation *GD* (either G″st or φ′st) computed after the creation of the barrier (i.e., for *T* > 0), we used the R‐package *randomForest* (Liaw & Wiener, 2002) to fit the model GD ~ *T* × *H_e_*. We used 200 trees and a sample size of 500, as these values provided very good accuracy (mean squared errors lower than 0.4%). Created *randomForest* R‐objects were saved in the form of*.rda* files (the usual file format for saving R‐objects) and were further used to predict the four possible expected measures of genetic differentiation GD_max_ (two possible metrics of genetic differentiation and two possible mutation rates) between pairs of populations according to both the mean expected heterozygosity *H_e_* (the proxy for effective population sizes) and the number of generations *T* elapsed since barrier creation, using the *predict.randomForest* function.

The 20,460 simulations associated with *T* ≤ 0 (i.e., before the creation of the barrier) were used to predict the four possible measures of genetic differentiation GD_min_ (background signal) that may be expected under the influence of mutations, drift, and incomplete genetic sampling between two adjacent populations not separated by any barrier to gene flow. For each of both mutation rates *µ* and each of both metrics of genetic differentiation *GD* (either G″st or φ′st) computed before the creation of the barrier (i.e., for *T* < 0), GD_min_ was computed as the fifth percentile of non‐null simulated *GD* values. These four predicted GD_min_ values were stored in the form of a*.rda* file.

### 2.3 Computing the genetic index of fragmentation *F*_INDEX_

Equation 1 allows computing a unique index of fragmentation for each combination of both a mutation rate *µ* (5 × 10^–5^ or 5 × 10^–4^) and a metric of genetic differentiation *GD* (G″st or φ′st). The four indices are then averaged to get the final index of fragmentation *F*_INDEX_ with a 95% confidence interval computed as 1.96 × SE, with *SE* the estimated standard error (i.e., the estimated standard deviation divided by $\sqrt{4}$).

Note that when several genotypic datasets are available for the same obstacle, for instance when several sympatric species are sampled on either side of the obstacle or when several replicates are considered (as is the case of all simulated data in this study), an overall *F*_INDEX_ can also be estimated using an intercept‐only mixed‐effect linear model with the various indices as the response variable and the genotypic dataset as a random effect (Bates, Mächler, Bolker, & Walker, 2015). This procedure allows taking into account the fact that *F*_INDEX_ values computed from the same dataset are not independent and thus avoids biased estimates of standard errors *SE* (McNeish, 2014). The overall *F*_INDEX_ is obtained from the estimated intercept of the model (which simply amounts to calculating the average of indices across datasets), and the corresponding 95% confidence interval is computed as 1.96 × SE, with *SE* the unbiased standard error as estimated from the mixed‐effect model.

**Figure 1:**
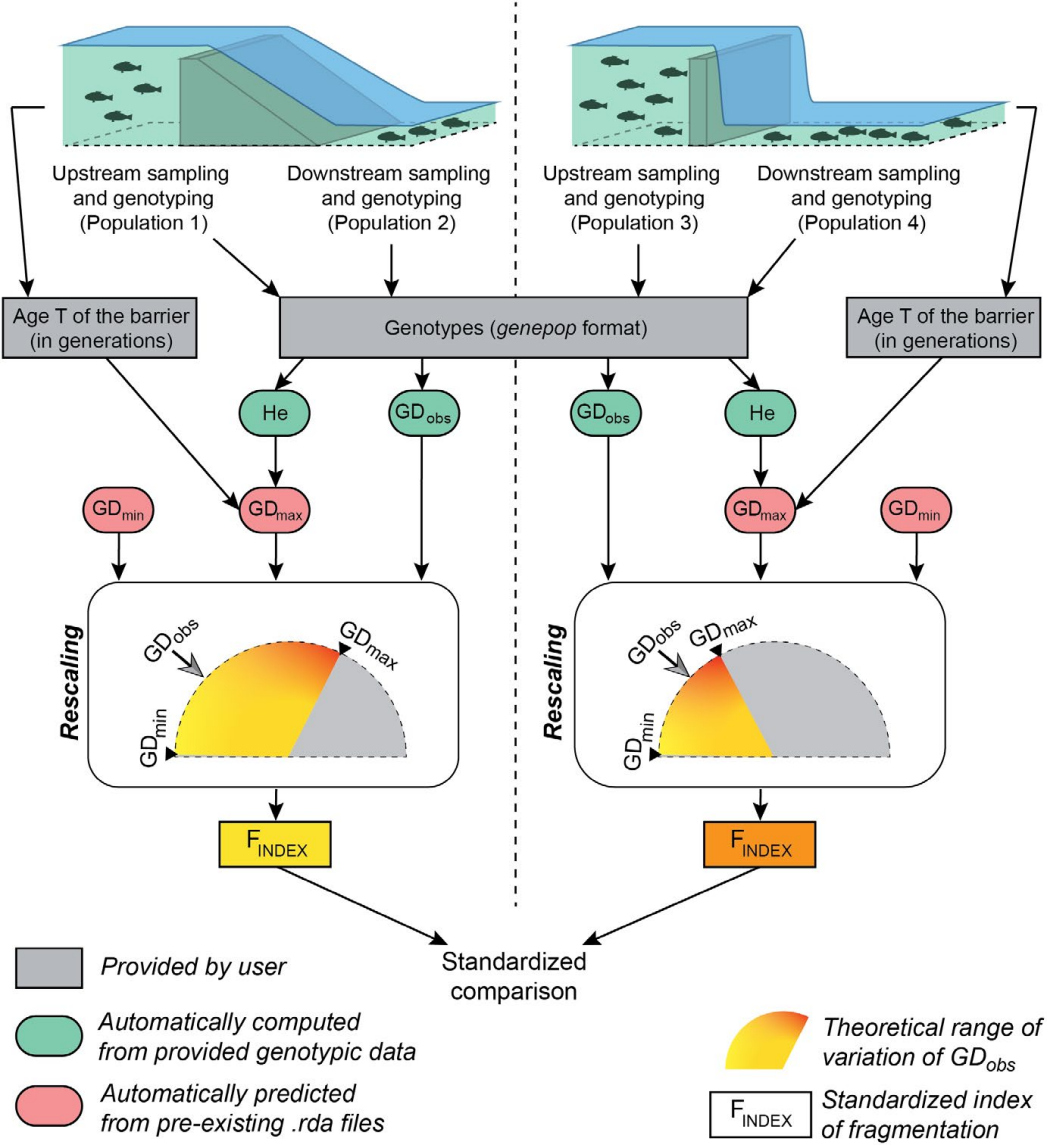
Flowchart illustrating the major steps in calculating the genetic index of fragmentation for two independent obstacles. This flowchart refers to a user‐friendly script made publicly available. After the sampling of populations located at the immediate upstream and downstream vicinity of each obstacle, users only have to provide a file of genotypes in the *genepop* format and a file of parameters indicating, for each obstacle, the names of the sampled populations and the number *T* of generations elapsed since the creation of the obstacle. Observed measures of genetic differentiation GD_obs_ and mean expected heterozygosity *H_e_* are automatically computed from provided genotypic data. GD_min_ and GD_max_ values, both delimiting the theoretical range of variation of GD_obs_, are automatically predicted from pre‐existing.rda files, GD_max_ values depending on both *H_e_* and *T*. The computation of the index basically amounts to rescaling GD_obs_ within its theoretical range (see main text for details), thus allowing standardized comparisons of the permeability of various obstacles, whatever their age, the considered species or the effective size of sampled populations

The whole procedure was automated within a user‐friendly R‐function (the FINDEX R‐function; see Appendix S1). Users are simply expected to provide empirical genotypic datasets (in the *genepop* format) and a parameter file indicating for each considered obstacle the name of the two adjacent populations (as given in the genotypic datasets) and the number of generations elapsed since barrier creation. This number of generations is to be estimated from the life‐history traits of the considered species. Figure 1 provides a flowchart allowing an overall visualization of the process.

### 2.4 Validation of the *F*_INDEX_ from simulated data

To assess the validity of the proposed *F*_INDEX_ in response to different levels of obstacle permeability, we again used the program QuantiNemo2 to simulate gene flow over 1,000 nonoverlapping generations between two adjacent demes of constant carrying capacity *K*, with *K = *50, 100, 250, 500, or 1,000 individuals. To mimic realistic genetic datasets, each microsatellite locus was given a unique stepwise mutation rate *µ* randomly picked from a log‐normal distribution ranging from 5 × 10^–5^ to 5 × 10^–3^ with a mean of 5 × 10^–4^ (see Appendix S7 for details). The inter‐deme migration rate was set to 0.5 for the first 400 generations and then dropped to *m* for the last 600 generations, with *m* ranging from 0 to 0.2 with an increment of 0.01 and from 0.2 to 0.5 with an increment of 0.05, mimicking the creation of a more or less severe barrier to gene flow (total barrier, crossing rate *m* = 0; no barrier, crossing rate *m* = 0.5). All other simulation parameters were similar to previous simulations. For each carrying capacity *K* and each crossing rate *m*, we ran 20 simulation replicates, and 30 genotypes were sampled at generations *t* = 405 (age of the barrier *T* = 5), 410, 415, 420, 425, 450, 500, and 700 (*T* = 300), resulting in a total of 21,600 simulated genetic datasets in the *Fstat* format, further converted into the *genepop* format.

For each simulated dataset, we computed the averaged expected heterozygosity *H_e_* and the two pairwise measures of genetic differentiation G″st and φ′st. We then used parameters *T* and *H_e_* to predict the corresponding measures of genetic differentiation GD_min_ and GD_max_ (for both G″st and φ′st) expected under the two mutation rates 5 × 10^–5^ and 5 × 10^–4^ using the *predict.randomForest* function and the previously created*.rda* files (Appendix S1). For each dataset, the four indices of fragmentation were then computed using Equation 1. To average datasets across replicates, we finally used intercept‐only mixed‐effect models (with dataset as a random effect) to get the final mean *F*_INDEX_ (along with a 95% confidence interval) corresponding to each combination of *K*, *T,* and *m*.

We finally explored the sensitivity of the *F*_INDEX_ to uncertainty in the estimates of *N_e_* and *T* and to reduced numbers of markers. Details are provided in Appendices S12 to S14.

### 2.5 Test of the *F*_INDEX_with empirical data

To assess the behavior of the proposed *F*_INDEX_ in real situations, we used two published empirical datasets. The first one is from Gouskov, Reyes, Wirthner‐Bitterlin, and Vorburger (2016). In this study, authors assessed riverscape fragmentation induced by 37 hydroelectric recent power stations in the Rhine catchment using data from 2,133 European chubs (*Squalius cephalus*) sampled across 47 sites and genotyped at nine microsatellite loci. We selected 6 pairs of populations according to the following criteria: upstream and downstream populations belonged to the same river, were separated by a single dam, were distant from a maximum of 5km (the maximum migration distance observed in chubs being 16 km according to Fredrich, Ohmann, Curio, & Kirschbaum, 2003), and were not separated by any confluence with important tributaries. This selection corresponded to 6 independent dams created between 1893 and 1964 (~4 to 10 meters high), all equipped with a fishpass (Table 1; see also Appendix S8 for a map). We considered a generation time of 3 years, as reported in Gouskov et al. (2016) to compute the number of generations elapsed since barrier creation and ran the developed FINDEX R‐function (Appendix S1) to automatically compute *F*_INDEX_ values.

**Table 1:** Main characteristics and results for the obstacles selected from empirical datasets (Original publication: (1) Gouskov et al., 2016; (2) Prunier et al., 2018)

River	Obstacle	Creation date	Upstream–Downstream distance (km)	Species	Number of elapsed generations	*H_e_*	*F*_INDEX_	95%CI	Original publication
**Rhine**	**Barr11**	**1964**	**4.79**	***Sc***	**15.33**	**0.69**	**55.22**	**5.71**	**(1)**
**Aar**	**Barr13**	**1902**	**1.91**	***Sc***	**36.00**	**0.76**	**49.53**	**10.72**	**(1)**
Aar	Barr17	1893	3.14	*Sc*	39.00	0.77	0	0	(1)
**Aar**	**Barr19**	**1896**	**1.93**	***Sc***	**38.00**	**0.76**	**61.87**	**3.16**	**(1)**
Aar	Barr26	1963	4.84	*Sc*	15.67	0.75	0	0	(1)
Limmat	Barr33	1933	3.24	*Sc*	25.67	0.72	0	0	(1)
**Célé**	**CLA**	**1500**	**0.18**	***Go***	**204**	**0.60**	**42.53**	**3.88**	**(2)**
**Célé**	**SCA**	**1500**	**0.09**	***Go***	**204**	**0.63**	**35.09**	**2.60**	**(2)**
**Célé**	**SCC**	**1960**	**0.2**	***Go***	**20**	**0.64**	**64.97**	**2.81**	**(2)**
Viaur	SEG	1600	0.11	*Go*	164	0.58	6.90	4.33	(2)
Viaur	CAM	1600	0.49	*Go*	164	0.62	0	0	(2)
**Viaur**	**CAP**	**1700**	**0.55**	***Go***	**124**	**0.61**	**42.22**	**0.97**	**(2)**
**Viaur**	**SJU**	**1800**	**1.07**	***Go***	**64**	**0.62**	**45.01**	**3.16**	**(2)**
**Viaur**	**CIR**	**1960**	**0.97**	***Go***	**20**	**0.62**	**37.55**	**15.60**	**(2)**
**Célé**	**CLA**	**1500**	**0.18**	***Pp***	**255**	**0.54**	**36.12**	**3.80**	**(2)**
Célé	SCA	1500	0.09	*Pp*	255	0.57	0	0	(2)
Célé	SCC	1960	0.2	*Pp*	25	0.58	10.05	11.41	(2)
Viaur	SEG	1600	0.11	*Pp*	205	0.63	12.03	13.61	(2)
Viaur	CAM	1600	0.49	*Pp*	205	0.61	0	0	(2)
Viaur	CAP	1700	0.55	*Pp*	155	0.67	0	0	(2)
Viaur	SJU	1800	1.07	*Pp*	105	0.70	0	0	(2)
Viaur	CIR	1960	0.97	*Pp*	25	0.70	0	0	(2)
**Célé**	**CLA**	**1500**	**0.18**	***Go‐Pp***	/	/	**39.32**	**6.23**	**(2)**
Célé	SCA	1500	0.09	*Go‐Pp*	/	/	17.55	34.39	(2)
Célé	SCC	1960	0.2	*Go‐Pp*	/	/	37.51	53.83	(2)
Viaur	SEG	1600	0.11	*Go‐Pp*	/	/	9.47	6.88	(2)
Viaur	CAM	1600	0.49	*Go‐Pp*	/	/	0	0	(2)
Viaur	CAP	1700	0.55	*Go‐Pp*	/	/	21.11	41.38	(2)
Viaur	SJU	1800	1.07	*Go‐Pp*	/	/	22.51	44.12	(2)
Viaur	CIR	1960	0.97	*Go‐Pp*	/	/	18.78	36.80	(2)

Note

For each obstacle, the table indicates the name of the river, the date of creation, the distance between upstream and downstream sampled populations, the considered species (*Sc*: *Squalius cephalus*; *Go*: *Gobio occitaniae*; *Pp*: *Phoxinus phoxinus*), the number of generations elapsed since barrier creation, the mean expected heterozygosity (*H_e_*), and the computed *F*_INDEX_ along with its 95% confidence interval. In bold, obstacles that were found as significant barriers to gene flow.

The second empirical dataset is from Prunier, Dubut, Loot, Tudesque, and Blanchet (2018). In this study, authors assessed the influence of various anthropogenic stressors including riverscape fragmentation induced by weirs on patterns of genetic diversity and differentiation in two freshwater fishes from two distinct rivers in southwestern France. They used data from 1361 Eurasian minnows (*Phoxinus phoxinus*) and 1359 Languedoc gudgeon (*Gobio occitaniae*) sampled across 47 sites (22 in the Célé River and 25 in the Viaur River) and genotyped at 11 and 13 microsatellite loci, respectively. We selected 8 pairs of populations according to the following criteria: upstream and downstream populations belonged to the same river, were separated by a single weir, were distant from a maximum of 1km, were not separated by any confluence with tributaries, and were sampled for both species. This selection corresponded to 8 independent weirs (~1 to 3 m high) created between the 16th and the 20th century (Table 1; see also Appendix S8 for maps). We considered a generation time of 2 years in *P. phoxinus* and 2.5 years in *G. occitaniae* to compute the number of generations elapsed since barrier creation and again ran the FINDEX R‐function (Appendix S1) to automatically compute *F*_INDEX_ values for each obstacle, each species and across species.

## 3 RESULTS

### 3.1 Expected measures of genetic differentiation

**Figure 2:**
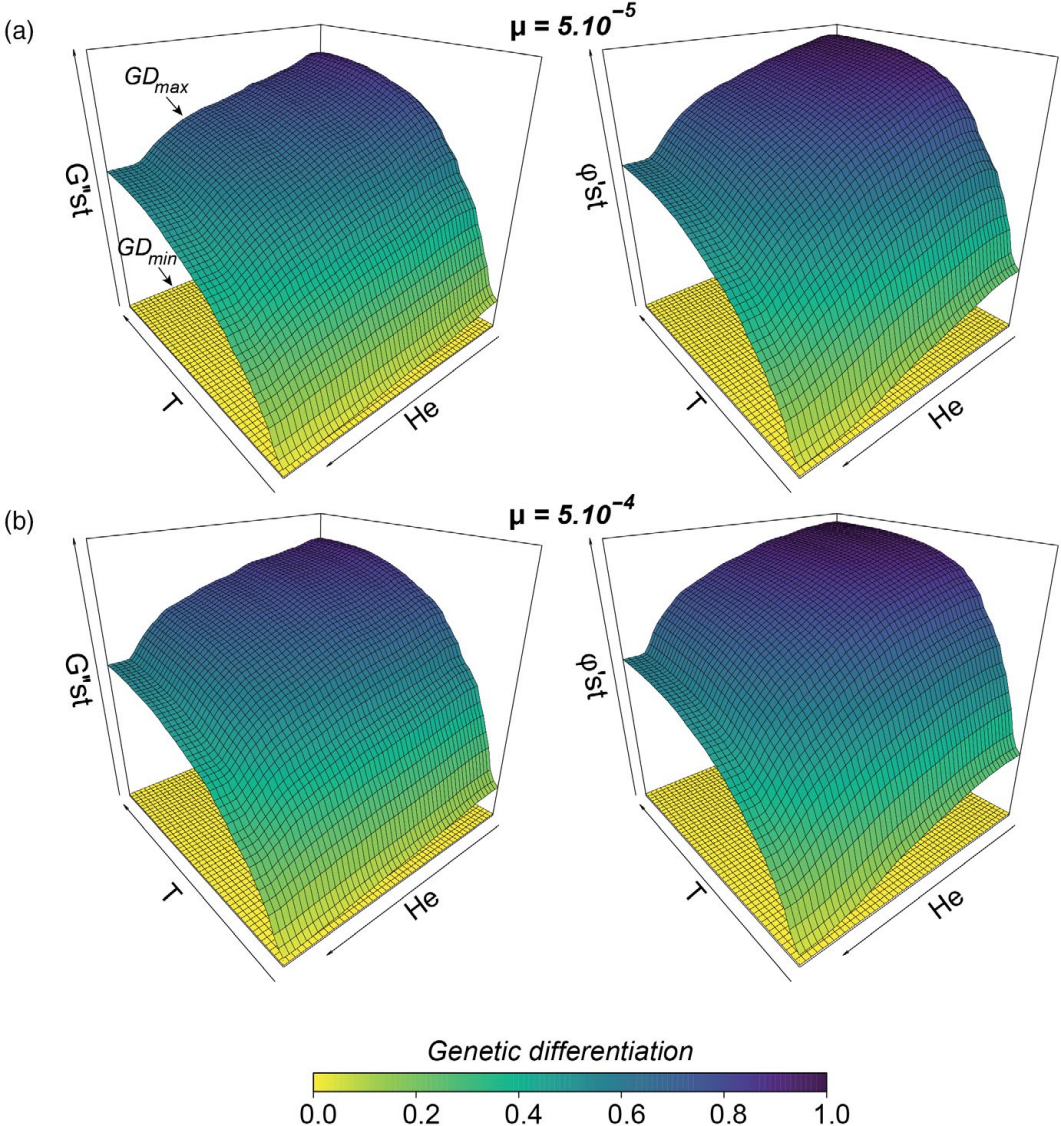
For each mutation rate (panels A and B) and each metric of genetic differentiation (G″st on the left and φ′st on the right), predicted GD_max_ variations across the parameter space defined by the number *T* of generations elapsed since total barrier creation (from 0 to 600 generations) and the averaged expected heterozygosity (*H_e_*, ranging from 0 to 0.93) for pairs of adjacent populations. GD_min_ values are represented at the bottom of each graph. GD_min_ and GD_max_ surfaces together delimit the theoretical range of variation for any observed measure of genetic differentiation GD_obs_

The first set of simulations was designed to predict GD_min_ and GD_max_ values, that is, the lower and upper limits of the theoretical range of variation of GD_obs_. Data simulated before the creation of the barrier (*m* = 0.5; *t* < 400; *T* < 0) were used to predict GD_min_ values whereas data simulated after the creation of the barrier (*m* = 0; *t* ≥ 400; *T* ≥ 0) were used to predict GD_max_ values. As expected with a migration rate of 0.5, GD_min_ values were always very close from 0 (~0.8 × 10^–3^ for G″st, ~1.2 × 10^–3^ for φ′st; Appendix S6). These values represent the predicted background levels of genetic differentiation resulting from the sole influences of random processes such as genetic drift, mutations, and sampling biases (Figure 2).

GD_max_ values were on the contrary designed to mimic the temporal inertia in the setting up of genetic differentiation after the creation of a total barrier to gene flow. They were predicted from the number T of elapsed generations since barrier creation and the averaged expected heterozygosity *H_e_* from simulated data using a random forest algorithm. With explained variance ranging from 86.8% to 94.2%, random forest models accurately captured variations in measures of genetic differentiation across the parameter space, whatever the considered mutation rate or the considered metric of genetic differentiation (see Appendices S9 and S10). As expected in absence of gene flow (Figure 2), GD_max_ increased with time since barrier creation and decreased with the increase in effective population size (i.e., with *H_e_*). With predicted GD_max_ values ranging from 0.031 to 0.898 for G″st and from 0.042 to 0.968 for φ′st, both metrics displayed similar distribution patterns across mutation rates, although φ′st systematically showed higher values at low *H_e_*.

### 3.2 Validation of the *F*_INDEX_from simulated data

**Figure 3:**
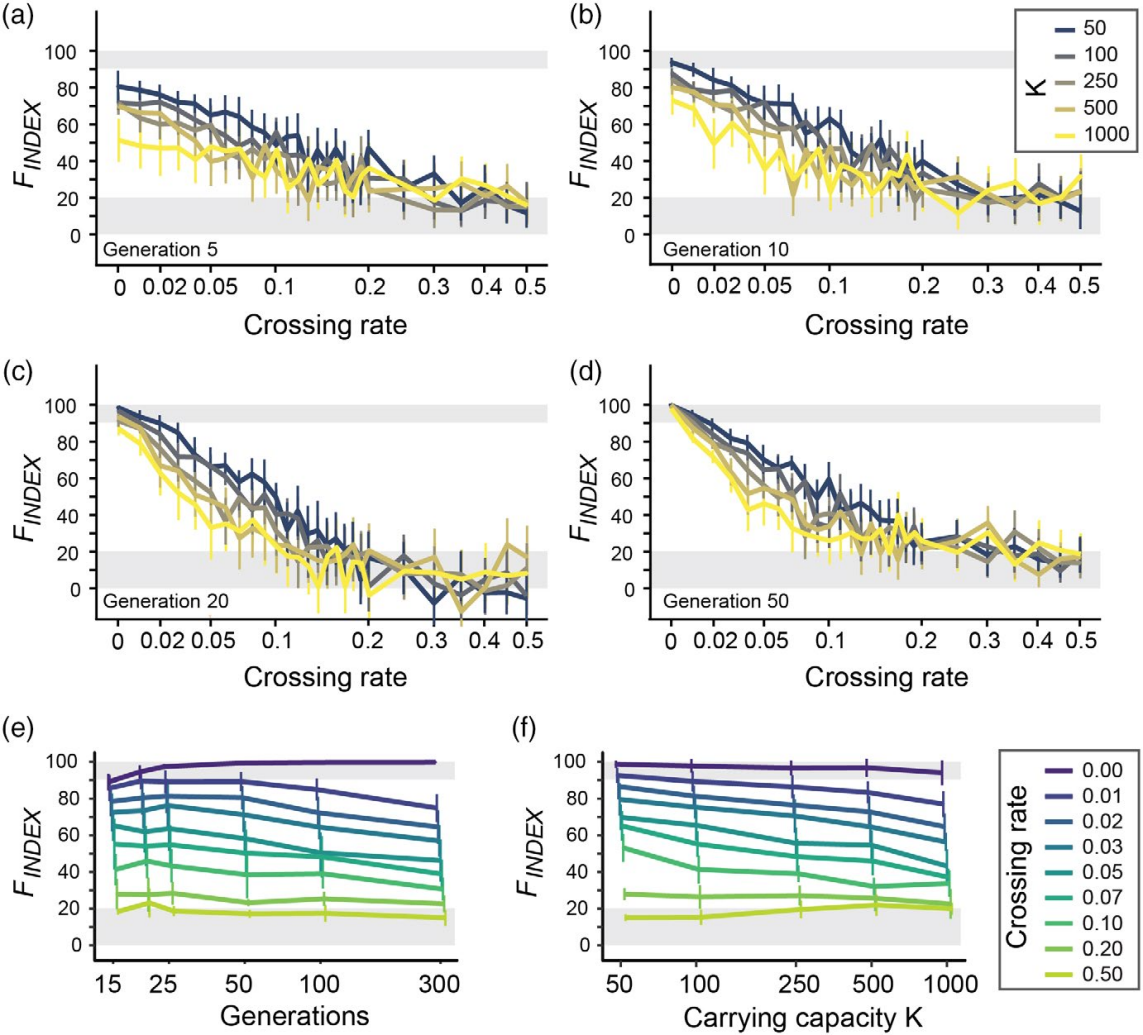
Panels a–d: *F*_INDEX_responses to the increase in crossing rate (*m*, on a logarithmic scale) for five different carrying capacities *K* (colored lines) and from 5 to 50 generations after barrier creation. Results for a number of generations higher than 50 are visually similar to panel d (not shown; but see panel e). All *F*_INDEX_ values were averaged over 20 simulated replicates and plotted with 95% confidence intervals. Panels e, f: *F*_INDEX_responses to the increase in time since barrier creation (panel e) and to the increase in carrying capacity *K* (panel f) for eight different crossing rates *m* (colored lines). The mean *F*_INDEX_ values computed over simulated replicates were here averaged over carrying capacities (panel e) or over generations (excluding generations ≤ 10; panel f) and plotted with standard deviations. In all panels, shaded gray areas represent the ranges of variations in which the monitored obstacle can be considered as acting as a total barrier to gene flow (*F*_INDEX_ > 90%) or, on the contrary, as allowing full genetic connectivity (*F*_INDEX_ < 20%)

The second set of simulations was designed to assess whether the *F*_INDEX_ correctly reflected the actual level of gene flow between two populations separated by an artificial barrier, beyond the temporal inertia in the setting up of genetic differentiation. The mean *F*_INDEX_ values computed over simulated replicates for each combination of *K* (carrying capacity), *T* (number of generations since barrier creation), and *m* (obstacle crossing rate) showed—as expected—an overall decrease with the increase in crossing rate, whatever the size of populations or the age of the barrier (Figure 3a–d). As expected when population are connected with high crossing rates (*m* > 0.2, a crossing rate of 0.5 leading to full connectivity), the 95% confidence intervals about the *F*_INDEX_ always included values lower than 20%. On the contrary, in absence of gene flow (*m* = 0), the 95% confidence intervals always included values higher than 90%, except within the first 10 generations after barrier creation (Figure 3a, b). In these cases, the *F*_INDEX_was slightly biased downwards, which indicates that we could not totally rule out the noise associated with the measurement of genetic differentiation within the 10 first generations after barrier creation (Appendix S11). Nevertheless, the *F*_INDEX_showed valid and consistent values for both lowest and highest crossing rates, the two thresholds of 90% (total barrier to gene flow) and 20% (full gene flow) providing robust benchmarks for future interpretation of the index, whatever the age of the obstacle (from generation 10 at least) or the effective size of populations.

For low—though non‐null—crossing rates (0 < *m ≤ *0.1), the *F*_INDEX_showed higher variability, with two noticeable trends. First, whatever the simulated carrying capacity, the *F*_INDEX_showed a slight 10% to 20% decrease with the increase in time since barrier creation (from generation 15 to generation 300; Figure 3e). For a crossing rate of *m* = 0.05 for instance, *F*_INDEX_ values decreased from 65% at generation 15 to ~46% at generation 300. Secondly, whatever the generation (>10), the *F*_INDEX_showed a slight 10 to 30% decrease with the increase in effective population sizes (from carrying capacity *K* = 50 to 1,000; Figure 3f). For a crossing rate of *m* = 0.05 for instance, *F*_INDEX_ values decreased from 70% in smallest populations (*K* = 50) to ~ 43% in largest populations (*K* = 1,000).

Sensitivity analyses showed that the *F*_INDEX_ is highly robust to a ~50% uncertainty in the estimates of *T* (Appendix S12) and that 95% CI about *F*_INDEX_ values correctly capture uncertainty associated with the use of *H_e_* as a proxy for *N_e_* (Appendix S13). Finally, we found that the *F*_INDEX_ is highly robust to a limited number of microsatellite markers, but tends to slightly underestimate barriers effects when using low polymorphic markers (Appendix S14).

### 3.3 Test of the *F*_INDEX_with empirical data

**Figure 4:**
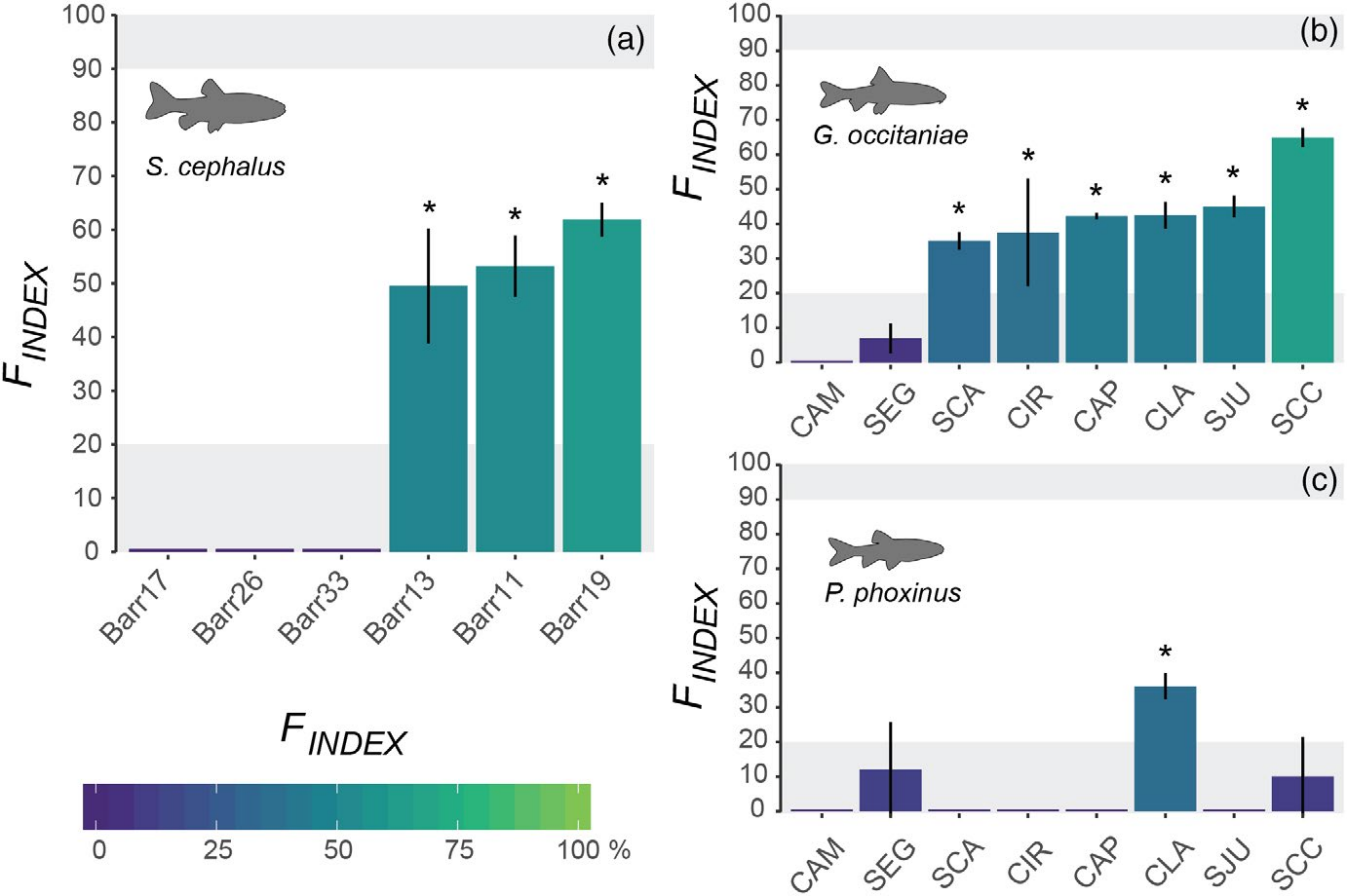
*F*_INDEX_ values and associated 95% confidence intervals as computed from empirical genetic datasets in chubs (panel a), gudgeons (panel b), and minnows (panel c). In all panels, shaded gray areas represent the ranges of variations in which the monitored obstacle can be considered as acting as a total barrier to gene flow (*F*_INDEX_ > 90%) or, on the contrary, as allowing full genetic connectivity (*F*_INDEX_ < 20%). Stars indicate a significant barrier effect of the obstacle. See Table 1 for details

In the first empirical dataset (Gouskov et al., 2016), monitored dams were created from 1893 to 1964, which corresponds to ~15 to 39 generations in *S. cephalus* (Table 1). Averaged levels of expected heterozygosity were high and showed little variability (ranging from 0.69 to 0.77), whereas observed measures of genetic differentiation were pretty low, ranging from 0 to 0.028 for φ′st and from 0 to 0.025 for G″st. We found that three dams showed a *F*_INDEX_value ranging from 49% to 62%, suggesting a 49% to 62% local decrease in genetic connectivity (Figure 4a). The other three dams all showed null *F*_INDEX_values, indicating that populations located on either side of the barrier are fully connected by gene flow (Table 1). Importantly, *F*_INDEX_values were independent from both time since barrier creation (Spearman correlation test, *ρ* = 0.03, *p* = .95) and averaged expected heterozygosity (*ρ* = −0.03, *p* = .95).

In the second empirical dataset (Prunier et al., 2018), monitored weirs were built between the 16th and the 20th century, that is approximately from 20 to 204 generations in *G. occitaniae* and from 25 to 255 generations in *P. phoxinus*. As previously, averaged levels of expected heterozygosity were high and showed little variability (ranging from 0.58 to 0.72), whereas observed measures of genetic differentiation were pretty low, ranging from 0 to 0.034 for φ′st and from 0 to 0.026 for G″st. The impact of weirs was variable across space and species (Table 1; Figure 4b). In *G. occitaniae*, six weirs (out of eight) were found as responsible for a decrease in genetic connectivity since barrier creation (*F*_INDEX_ > 20%), with *F*_INDEX_ values ranging from 35% in the case of barrier SCA to 65% in the case of barrier SCC in the Célé River. In *P. phoxinus*, all weirs but CLA in the Célé River (*F*_INDEX_ = 36%) were found as highly permeable (*F*_INDEX_ < 20%), with five out of eight weirs showing a *F*_INDEX_of 0%. When computed across species, only the barrier CLA in the Célé River (multispecies *F*_INDEX_ = 39%) was identified as an obstacle to overall genetic connectivity (other *F*_INDEX_values ranging from 0% to 37.5%, with 95% confidence intervals systematically including the 20% threshold; Table 1). As previously, *F*_INDEX_values in each species were independent from both time since barrier creation (|*ρ|* < 0.46, *p* > .25) and averaged expected heterozygosity (|*ρ|* < 0.54, *p* > .16).

## 4 DISCUSSION

Restoring riverscape connectivity is of crucial importance in terms of biodiversity conservation, and it is now often subject to regulatory obligations (e.g., in Europe, the Water Framework Directive 2000/60/EC). However, rivers are subject to many and sometimes contradictory uses (Reid et al., 2018): For practitioners to be able to propose informed trade‐offs between restoring riverscape connectivity and maintaining infrastructures and their associated socioeconomic benefits (Hand et al., 2018; Roy et al., 2018; Song et al., 2019), new tools have to be developed, allowing a rapid and reliable quantification of the relative impacts of obstacles to freshwater species movements (see for instance Escoda, Fernández‐González, & Castresana, 2019). Our objective was here develop an operational tool allowing such thorough quantification from a minimum amount of fieldwork and data (Figure 1; see Box 1 for user guidelines).

The proposed genetic index of fragmentation *F*_INDEX_ can be easily and automatically computed from a simple set of upstream and downstream genotypes collected once and in the direct vicinity of a putative barrier, provided the approximate number of generations elapsed since barrier creation is known (the *F*_INDEX_ being highly robust to uncertainty in time since barrier creation; Appendix S13). Based on two complementary metrics of genetic differentiation (G″st and φ′st) preliminary chosen so as to limit any possible bias, the *F*_INDEX_ simply scales the observed level of genetic differentiation (GD_obs_) with respect to a theoretical range of variation spanning from the background noise expected in the absence of any barrier to gene flow (GD_min_ ~ 0, *F*_INDEX_ = 0%) to the maximal level of differentiation expected if the obstacle was a total barrier to gene flow (GD_max_, *F*_INDEX_ = 100%). The latter takes into account both the time since barrier creation and, using *H_e_* as a proxy, the effective size of populations, which makes the *F*_INDEX_ a truly innovative tool as it makes it possible to compare the actual barrier effect of obstacles differing by their age and/or by the size of the populations they separate. Using numerous simulations, we were able to obtain GD_max_ values for a large range of biologically realistic parameters (Figure 2). As expected, GD_max_ values progressively increased with time since barrier creation and decreased with the increase in averaged expected heterozygosity. Mutation rate also influenced GD_max_ patterns: As expected, higher mutation rates accelerate genetic differentiation through time when population sizes are small to medium. The use of two realistic mutation rates in GD_max_ predictions allows taking into consideration uncertainty in our proxy for effective population sizes, in the form of a 95% confidence interval about *F*_INDEX_ values (see Appendices S5b and S12).

The *F*_INDEX_ showed constant patterns of decrease with the increase in crossing rates (from *m* = 0 to *m* = 0.2), whatever the number of generations since barrier creation and the effective population size (Figure 3). For the lowest crossing rates (*m* ≤ 0.05), we found, however, that it could underestimate barrier effects in the first 5 to 10 generations after the creation of the obstacle. As a conservative strategy, we suggest that the *F*_INDEX_ should not be used to assess the permeability of obstacles separating populations for fewer than 10 generations. However, it is noteworthy that the *F*_INDEX_ can be applied to any type of organisms and thus that species with short generation time (such as some invertebrate species) may be considered as good candidates to investigate the impact of recently built barriers (e.g., <10 years ago). For the lowest crossing rates (*m* ≤ 0.1), we also found that *F*_INDEX_ values slightly decreased with both time since barrier creation (from generations 15 to 300) and effective population sizes (from simulated carrying capacities *K* = 50 to *K* = 1,000; Figure 3e, f). These trends have to be kept in mind when comparing intermediate *F*_INDEX_ values ranging from ~40 to ~80% (see Box 1 for guidelines).

Box 1 **Guidelines for the use and the interpretation of the *F*_INDEX_**

The *F*_INDEX_ allows an individual and standardized quantification of the impact of artificial barriers on riverscape functional connectivity from snapshot measures of genetic differentiation. Here, we provide a guideline for practitioners:

- **Species**: Any freshwater species whose local effective population sizes are lower than 1,000 can be considered.
- **Obstacle:** Any obstacle whose age corresponds to a minimum of 10–15 generations and a maximum of 600 generations for the studied species can be considered.
- **Sampling:** Populations are sampled in the immediate upstream and downstream vicinity of the obstacle, with a minimum of 20–30 individuals per population.
- **Genetic data**: Individual genotypes are based on a set of highly polymorphic microsatellite markers.
- **Computation:** The *F*_INDEX_ is computed in R thanks to a user‐friendly script made publicly available (see Data Archiving statement and Appendix S1 for a walkthrough).
- **Interpretation for** *F*_INDEX_ **> 90%:** A *F*_INDEX_ value higher than 90% (or whose 95% confidence interval includes the 90% threshold) indicates no gene flow between populations (total barrier effect), whatever the age of the obstacle or the effective size of populations.
- **Interpretation for** *F*_INDEX_ < **20%:** A *F*_INDEX_ value lower than 20% (or whose 95% confidence interval includes the 20% threshold) indicates full genetic connectivity (no barrier effect), whatever the age of the obstacle or the effective size of populations.
- **Interpretation for intermediate** *F*_INDEX_ **values:** Intermediate *F*_INDEX_ values can be used to rank obstacles according to their barrier effect. However, for *F*_INDEX_ values ranging from ~40 to ~80%, the *F*_INDEX_ tends to slightly decrease with both the increase in the number of generations since barrier creation and the increase in effective population sizes (Figure 3e, f). Obstacles with *F*_INDEX_ values that do not differ by more than 15 to 20% but that are characterized by very different ages and/ or population sizes (as indicated for instance by large differences in expected heterozygosity) should be considered as possibly having comparable barrier effects, except of course if the ranking of obstacles based on *F*_INDEX_ values goes against these trends. Consider for instance an obstacle A of age 20 (in generations) and an obstacle B of age 300. If *F*_INDEX_(A) = 40% and *F*_INDEX_(B) = 20%, both obstacles should be considered as possibly having the same impact on gene flow. On the contrary, if *F*_INDEX_(A) = 20% and *F*_INDEX_(B) = 40%, obstacle B can be confidently considered as more impactful than obstacle A. Similarly, consider an obstacle C separating populations with low expected heterozygosity (suggesting small effective population size) and an obstacle D separating populations with high expected heterozygosity. If *F*_INDEX_(C) = 40% and *F*_INDEX_(D) = 20%, both obstacles should be considered as possibly having the same impact on gene flow. On the contrary, if *F*_INDEX_(C) = 20% and *F*_INDEX_(D) = 40%, obstacle D can be confidently considered as more impactful than obstacle C.

Nevertheless, the *F*_INDEX_ provides a promising individual quantification of both the short‐ and long‐term genetic effects of dam‐induced fragmentation, allowing robust comparisons among species or populations with different population sizes and obstacles of different ages (from generation 15 at least) and types. When applied to empirical data, the *F*_INDEX_ allowed identifying several obstacles partially limiting gene flow in the three considered freshwater fish species (Figure 4). In each dataset, computed *F*_INDEX_ values were systematically independent from both time since barrier creation and averaged expected heterozygosity, indicating that the *F*_INDEX_ properly takes into account the differential evolution of allelic frequencies on either side of the barrier. Interestingly, the SCC weir on the Célé River (Prunier et al., 2018) showed contrasting results in gudgeons and minnows: It was identified as the most impactful obstacle in gudgeons (*F*_INDEX_ = 65%; Table 1) whereas it was found as highly permeable to gene flow in minnows (*F*_INDEX_ = 10%). More generally, minnows were much less affected by obstacles than gudgeons, in accordance with personal field observations and previous findings on the same two rivers (but from independent datasets; Blanchet et al., 2010). Although understanding how obstacle typological features (height, slope, presence of a secondary channel, etc.; Baudoin et al., 2014) and fish traits (body size, movement capacities, etc.; Blanchet et al., 2010) might interact and shape riverscape patterns of functional connectivity was beyond the scope of this study, these results suggest that future comparative studies based on the proposed *F*_INDEX_ might provide thorough insights as to the determinants of dam‐induced fragmentation in various freshwater organisms (Richardson, Brady, Wang, & Spear, 2016), including fish but also other taxa such as macro‐invertebrates that display very contrasting traits related to dispersal (e.g., Alp, Keller, Westram, & Robinson, 2012).

Despite its strong operational potential, the *F*_INDEX_, however, does not come without some limitations (see Box 2 for a list of possible future developments). First of all, it is important to remember that this index is a measure of genetic connectivity, not demographic connectivity (Lowe & Allendorf, 2010), and thus cannot directly provide any counting of the actual number of crossing events. If immigrants do not reproduce, the actual crossing of dozens of individuals, although suggesting high permeability, might not translate into low *F*_INDEX_ values. Although this is more of an inherent characteristic of the index than a real limitation, it is important to keep this specificity in mind when interpreting it. Furthermore, a crossing rate has to be interpreted in regard of effective population sizes: A crossing rate of 0.05 actually corresponds to 2.5 effective dispersal events per generation in populations of size 50, but to 50 effective dispersal events in populations of size 1,000. This higher permeability in the latter case translates into *F*_INDEX_ values being systematically slightly lower when simulated population sizes are larger (at a given intermediate crossing rate; Figure 3f). However, since the actual effective size of natural populations is generally unknown, these differences in *F*_INDEX_ values may be difficult to interpret when handling empirical data. We provide guidelines for the interpretation of *F*_INDEX_ values in Box 1.

Box 2 **Future directions for improving the *F*_INDEX_**

The *F*_INDEX_ is already operational but it is, however, still in its infancy. We identified several research avenues that may allow further improving it or help answer specific needs. They are here presented by our order of priority.

- **Taking asymmetric crossing into consideration:** The proposed *F*_INDEX_ currently relies on the use of classical pairwise measures of genetic differentiation that assume symmetric gene flow. It will be first necessary to assess the sensitivity of the current version of the *F*_INDEX_ to asymmetric barrier effects and, if needed, to determine whether existing asymmetric measures of genetic differentiation (Sundqvist, Keenan, Zackrisson, Prodöhl, & Kleinhans, 2016) could be used to improve its efficiency. This task may otherwise require the development of new metrics of genetic differentiation.
- **Dealing with nonadjacent sampling designs:** The proposed *F*_INDEX_ relies on a strict *adjacent sampling strategy*, with populations sampled in the immediate upstream and downstream vicinity of the considered obstacle. However, this sampling design might be difficult to implement in some situations (e.g., dams with a large reservoir). When the two sampled populations are distant from each other, GD_obs_ values may nevertheless result from the interplay between the actual barrier effect (the quantity of interest) and other processes such as Isolation‐by‐Distance. In such situations, the *F*_INDEX_ should be computed using ad hoc GD_min_ and GD_max_. values, both taking into account the additional processes responsible for GD_obs_ values. To that aim, a solution could be to consider a *space‐for‐time substitution sampling design* (Coleman et al., 2018), with the additional sampling of (at least) two control populations that are not disconnected by any barrier, are located within the same river stretch and are separated by approximately the same distance as the two focal populations. Measures of genetic differentiation computed between these control populations could be directly used as new ad hoc GD_min_ values. An empirical migration rate *m* could then be inferred from these control measures of genetic differentiation, for instance using an Approximate Bayesian Computation approach (Bertorelle, Benazzo, & Mona, 2010; Csilléry, Blum, Gaggiotti, & François, 2010) and new genetic simulations be performed using *m* as the baseline migration rate, so as to get ad hoc GD_max_ values (taking into consideration the age of the obstacle and the effective size of focal populations). Such a procedure might help answer very specific needs, but its complexity might restrict the direct use of the *F*_INDEX_ to informed and trained managers. Furthermore, additional work would be required to determine to what extent *F*_INDEX_ values computed in that way could still be comparable across obstacles.
- **Handling other genetic markers:** The proposed *F*_INDEX_ relies on the use of microsatellite markers. Microsatellite markers are still widely used in the study of nonhuman organisms, especially by environmental managers. However, the reduction in sequencing costs and the development of an ever‐increasing supply of biotechnological services (Davey et al., 2011) now allow easier access to new genetic markers such as SNPs. In the future, a new version of the *F*_INDEX_ allowing the use of SNPs could be developed: It would require identifying relevant SNP‐based measures of genetic differentiation (see Appendices S3 and S4) as well as numerous simulations to establish theoretical distributions of GD_max_ values according to both time since barrier creation and expected heterozygosity.

Secondly, the computation of the *F*_INDEX_ relies on the assumption that, beyond the background signal of genetic differentiation that is expected under the sole influences of genetic drift, mutations and incomplete genetic sampling (GD_min_), the observed measures of genetic differentiation GD_obs_ only stem from dam‐induced fragmentation. In other words, it is crucial to consider situations in which the focal populations would be fully connected if the obstacle did not exist. This assumption is true only when sampled populations are adjacent, that is, located in the immediate upstream and downstream vicinity of the obstacle (Figure 1). Although restrictive, this adjacent sampling design has the advantage of making the *F*_INDEX_ valid for almost any freshwater species, regardless of their life‐history traits: The effective population size, a key parameter that may obviously differ across species, is indeed directly taken into consideration in the *F*_INDEX_computation, while differences in dispersal abilities can be considered as null at very short distances. It yet implies the exclusion of migratory fish species, though at the heart of great conservation issues (Junge et al., 2014; Klütsch et al., 2019): complex life cycles such as anadromy (“river‐sea‐river” migrations), catadromy (“sea‐river‐sea” migrations), or potamodromy (“river‐lake‐river” migrations) indeed preclude the delineation of upstream and downstream populations and do not allow proper estimates for the *F*_INDEX_. In nonmigratory fish species, this assumption also prevents the use of the *F*_INDEX_ in large‐scale studies, in which the distance between the upstream and the downstream sampling sites lies beyond the dispersal capacities of the studied species. It certainly leaves room for maneuver, as illustrated with the empirical dataset from Gouskov et al. (2016): We could for instance select pairs of populations located up to 5 km apart, but this was only possible because of the high mobility of chubs, and performed in an illustrative purpose: A maximum distance of 1km would have been safer. In low‐mobility species, a nonadjacent sampling design might bias the *F*_INDEX_ upwards and hence overestimate the effect of obstacles, as observed measures of genetic differentiation would result from dam‐induced fragmentation but also from other processes such as Isolation‐by‐Distance (Coleman et al., 2018). We thus strongly encourage practitioners to consider an adjacent sampling design as often as possible, although we readily acknowledge that this may not always be an easy task given safety and accessibility considerations. Furthermore, fish may not always be found in the direct vicinity of obstacles. For instance, the conversion of a river into a reservoir after the creation of a dam often leads to major habitat modification and shifts in species composition (Bednarek, 2001), which can force adapting the sampling design. A solution might be to capture the resultant background signal of genetic differentiation by simulating ad hoc GD_min_ and GD_max_ values under various scenarios of isolation (Isolation‐by‐Distance, Isolation‐by‐Resistance, etc.; McRae, 2006) in a way similar to the simulation of GD_max_ values in this study (Figure 2; see also Box 2). It is in this perspective that the provided R‐function already allows users to integrate their own GD_min_. and GD_max_. values (Appendix S1). However, we believe that the variety, the complexity, and the specificity of such scenarios would preclude the computation of standardized *F*_INDEX_ scores, comparable among obstacles, species, and studies. Although it might in some instances be considered a technical constraint, we argue that only a strict adjacent sampling design can warrant unbiased and reliable *F*_INDEX_ estimates.

Finally, the proposed *F*_INDEX_ does not take into account the possible asymmetric gene flow (and associated asymmetry in effective population sizes) created by barriers, as fish might struggle or even fail to ascent an obstacle (sometimes despite the presence of dedicated fishpasses; Silva et al., 2018) whereas dam discharge might on the contrary further increase or even force downstream movements (Pracheil et al., 2015). Although quantifying the asymmetric permeability of obstacles appears of crucial importance for informed conservation measures, the proposed *F*_INDEX_ currently relies on the use of classical pairwise measures of genetic differentiation that assume symmetric gene flow and similar effective population sizes on either side of an obstacle. This may for instance partly explain why we did not find any *F*_INDEX_ higher than 65% for weirs (Prunier et al., 2018) and 61% for dams (Gouskov et al., 2016; Table 1), a result that calls for future comparisons of the *F*_INDEX_ with direct monitoring methods (Cayuela et al., 2018). Future developments will be required to allow the *F*_INDEX_ to provide unbiased and distinct standardized scores for both upstream and downstream barrier effects (see Box 2). In the meanwhile, it may be interesting to also assess the validity of the *F*_INDEX_ in quantifying the effects of terrestrial obstacles, since asymmetric gene flow is not necessarily as pronounced as in river systems: Provided that populations are sampled in the direct vicinity of the obstacle, the *F*_INDEX_ might as well provide a standardized quantification of road‐induced fragmentation.

## 5 CONCLUSION

We here laid the groundwork for an operational tool dedicated to the individual and standardized quantification of the impact of artificial barriers on riverscape functional connectivity from measures of genetic differentiation. The proposed genetic index of fragmentation *F*_INDEX_ is designed to take into account the temporal inertia in the evolution of allelic frequencies resulting from the interplay between the age of the obstacle and the effective sizes of populations. Provided only adjacent populations are sampled, the *F*_INDEX_ allows a rapid and thorough ranking of obstacles only a few generations after their creation. The *F*_INDEX_ in its current form still suffers from some limitations, and it should be seen as the preliminary version of a future powerful bio‐indicator of habitat fragmentation, rather than as an end‐product. We call conservation and population geneticists to pursue the development of such an index, as we—as scientists—need to help managers resolve complex and urging social problems. In Box 2, we hence propose several research avenues. Nonetheless, the *F*_INDEX_ is robust, only requires a minimum amount of fieldwork and genotypic data and already solves several difficulties inherent to the study of dam‐induced fragmentation in river systems, making it a promising tool for the restoration of riverscape connectivity. The *F*_INDEX_ may allow practitioners to objectively identify obstacles that do not present any substantial conservation issue (from a connectivity perspective) and help them target their efforts and resources toward the most impactful ones. Similarly, it may allow tracking the expected temporal decrease in genetic differentiation after obstacle removal or fishpass setting and thus help evaluate the success of local mitigations and restoration measures in response to regulatory obligations. Finally, it might as well provide a standardized quantification of road‐induced fragmentation, a critical issue in terrestrial ecology.

## Supporting information

Appendix S1
Appendix S2
Appendix S3
Appendix S4
Appendix S5
Appendix S6
Appendix S7
Appendix S8
Appendix S9
Appendix S10
Appendix S11
Appendix S12
Appendix S13
Appendix S14

## Data Availability

The two simulated datasets as well as R‐objects allowing the computation of the *F*_INDEX_ (Prunier et al., 2019) are available at the Figshare Digital Repository (https://doi.org/10.6084/m9.figshare.9698879.v4) as well as on our personal website (http://www.jeromeprunier.fr/Tools.html).
